# Effects of psychosocial stimulation on improving home environment and child-rearing practices: results from a community-based trial among severely malnourished children in Bangladesh

**DOI:** 10.1186/1471-2458-12-622

**Published:** 2012-08-07

**Authors:** Baitun Nahar, Md Iqbal Hossain, Jena D Hamadani, Tahmeed Ahmed, Sally Grantham-McGregor, Lars-Ake Persson

**Affiliations:** 1 International Maternal and Child Health (IMCH), Department of Women’s and Children’s Health, Uppsala University, Akademiska sjukhuset, SE-751 85, Uppsala, Sweden; 2 International Centre for Diarrhoeal Disease Research, Bangladesh (ICDDR,B), Dhaka, Bangladesh; 3 Centre for International Health and Development (CIHD), Institute of Child Health, University College London, London, UK

**Keywords:** HOME, Child-rearing practices, Psychosocial stimulation, Food supplementation, Community-based intervention, Bangladesh

## Abstract

**Background:**

Parenting programmes are effective in enhancing parenting practices and child development. This study evaluated the effects of a intervention with psychosocial stimulation (PS) on the quality of the home environment and mothers’ child-rearing practices in a community-based trial with severely malnourished Bangladeshi children.

**Method:**

Severely underweight children (n = 507), 6–24 months of age, were randomly assigned to five groups: PS; food supplementation (FS); PS + FS; clinic-control (CC); and, hospital-control (CH). PS included fortnightly follow-up visits for six months at community clinics where a play leader demonstrated play activities and gave education on child development and child rearing practices. FS comprised cereal-based supplements (150–300 kcal/day) for three months. All groups received medical care, micronutrient supplements and growth monitoring. Mothers were given the Home Observation for Measurement of the Environment (HOME) inventory and a questionnaire on parenting at baseline and after six months to assess the outcome.

**Results:**

322 children completed the study. After six months of intervention the PS + FS and PS groups benefitted in the total HOME score (depending on the comparison group, effect sizes varied from 0.66 to 0.33 SD) The PS + FS and PS groups also benefitted in two HOME subscales: maternal involvement (effect sizes: 0.8 to 0.55 SD) and play materials, (effect sizes: 0.46 to 0.6 SD), and child-rearing practices scores (effect size: 1.5 to 1.1 SD). The PS + FS group benefitted 4.0 points in total HOME score compared with CH, 4.8 points compared with CC and 4.5 points compared with FS (p < 0.001 for all). The PS group benefitted 2.4 points compared with CH (p = 0.035), 3.3 points compared with CC (p = 0.004), and 2.9 points compared with FS (p = 0.006). Child-rearing practice scores of the PS + FS group improved 7.7, 6.4 and 6.6 points and the PS group improved 8.5, 7.2 and 7.4 points more than CH, CC and FS, respectively (p < 0.001 for all).

**Conclusions:**

Child-rearing practices of mothers of severely malnourished children and the quality of their home environment can be improved through community-based psychosocial stimulation with or without food supplementation. This may be of importance to promote child development.

## Background

Optimum child development and growth of young children needs mother/caregiver’s warmth and affection, sensitivity, responsiveness, cognitive stimulation as well as good nutrition and freedom from infection [1-3]. Any sustained disruption to this may result in irreversible damage to the child’s development, which may lead to difficulties at school and to behavioural, emotional and personality problems [4,5]. Improving parenting skills would be a cost-effective and sustainable strategy to promote child development [5].

In low-income countries young children are exposed to multiple developmental risk factors including poverty, malnutrition, poor stimulation at home, and lack of care that adversely affect the ability to reach their developmental potential [6]. Mothers/primary caregivers of malnourished children living in poor socioeconomic environments are often less sensitive to their child’s needs, less involved and emotionally responsive, and less engaged in reciprocal interaction with their children than caregivers of adequately or well-nourished children [7,8].

Parent education interventions have improved parenting practices and child development [9-11]. Home visiting interventions in Jamaica, Colombia and South Africa improved mother’s knowledge on child rearing [12], mother-child interaction [13], the home environment [14] and child development [12,14,15]. In two studies in Bangladesh, one used group sessions with mothers [16] whereas the other also used home visiting [17] and both studies showed improved knowledge of child rearing and higher scores on Home Observation for Measurement of the Environment (HOME) inventory.

In Bangladesh, malnutrition is one of the major health problems in children under five years of age [18] and there is a need for proper rehabilitation of these children. A recent case–control study conducted in Bangladesh found that the mothers of severely underweight children were younger, shorter, more frequently energy deficient and less educated compared with mothers of well-nourished children. The fathers were younger, poorer, less educated, and more likely to have low paid jobs [19]. Such characteristics are also likely to be associated with child care and parenting practices.

Little is known about parenting practices of Bangladeshi mothers. A survey among rural mothers found that almost half had no education and most are unaware of the importance of providing opportunities for play and conversation with their child [20].

A community-based trial with 5 groups including food supplementation (FS), psychosocial stimulation (PS), both treatments, community controls and hospital controls was carried out with severely underweight children aged 6–24 months (n = 507) attending the Dhaka hospital of International Centre for Diarrhoeal Disease Research, Bangladesh (ICDDR,B). The psychosocial stimulation included play activities and parenting education. After six months of intervention, children who had received any stimulation with or without food supplementation scored higher on the Mental Development Index (MDI) of the Bayley Scales of Infant Development (BSID-II) [21] (effect size 0.37 SD) and had a higher weight-for-age Z-score (WAZ) (effect size 0.26 SD). These results have been reported in a separate paper [22]. It is likely that improvements in mothers’ child rearing practices and the stimulation they provide in the home may have led to some of these benefits. In this paper, we report the effect of the interventions on the quality of home environment and child-rearing practices of the mothers of the same children six months after the start of intervention.

## Methods

Children aged 6–24 months with WAZ < −3SD who had recovered from acute infection at the hospital of ICDDR,B were the study participants. We assessed a total of 553 children for eligibility and enrolled 507 children in the randomised trial. Children with weight-for-length Z-score (WLZ) < −3SD, oedema, fever, congenital disorders, diseases affecting growth, no fixed residence or primary caregiver not capable to provide stimulation due to illness were excluded (n = 46). Four community nutrition follow-up units (CNFU) were established in the the residential areas from where most malnourished children attended Dhaka hospital of ICDDR,B.

### Randomisation

On discharge and after obtaining parental informed consent, the eligible children (n = 507) were randomly assigned to five groups: PS; FS; PS along with FS (PS + FS); clinic-control (CC) and hospital-control (CH). The CH group received fortnightly follow-up care at the hospital nutrition follow-up unit (HNFU) of ICDDR,B. The other 4 groups received fortnightly follow-up care at the CNFUs. All groups received similar basic care comprising growth monitoring, health education and micronutrient supplementation.

Randomisation was done by a researcher, who was not involved in the study. Four sets of separate randomisations were prepared for each CNFU, using a computer-generated, block-randomisation scheme, with permuted block lengths of 5 and 10.

#### Follow-up

Mothers/caregivers and their children were followed-up fortnightly at the assigned CNFU or HNFU for the first three months then monthly for the final three months. At each follow-up visit all groups received the following as a part of routine clinical management practiced at Dhaka Hospital of ICDDR,B [23];

##### Growth monitoring

Children’s weight, length/height, and mid-upper arm and head circumferences were measured according to standard procedures [24].

##### Health education

Lessons on primary health care were provided by the play leaders (PLs).

##### Micronutrient supplementation

At recruitment and during fortnightly follow up visits the children were supplied with multivitamin drops for a month (containing vitamin A, vitamin D, thiamin, riboflavin, pyridoxine, nicotinamide, calcium and ascorbic acid). They were also supplemented with zinc dispersible tablets and iron and folic acid tablets from 2–12 weeks after recruitment.

##### Others

All study children were immunised according to the Expanded Programme of Immunisation guidelines [25], and children older than one year were de-wormed with a single dose of albendazole, provided that they had not been treated in the previous six months.

### Interventions

#### Psychosocial stimulation

Trained female health workers (PLs) conducted sessions with *play activities* and *parenting education* for one hour with every child and mother of the PS and PS + FS groups. This was done at CNFU for six months; fortnightly for the first three months and then monthly for next three months i.e. 9 visits over 6 months.

##### Play sessions

The PL demonstrated play techniques with low-cost and culturally appropriate homemade toys and focused on helping the mothers to become more effective in teaching their children and enhancing maternal-child interactions. Mothers were encouraged to continue the activities at home. They were lent toys and picture books to take home and changed them at every visit for different toys.

##### Parenting education

The PL discussed early child development and the importance of mothers being responsive to their infants and playing, chatting, singing, showing love to and praising their child. They demonstrated how to chat and incorporate play into daily activities, such as feeding and bathing, in order to promote child development. The mothers were discouraged from using physical punishment. We also aimed to improve mothers’ self-esteem by praising them, listening to them and providing new skills. The sessions were participatory and mothers/caregivers were encouraged to share their views and suggestions.

#### Food supplementation

Children in the FS and PS + FS groups were supplemented with food packets for the first three months of follow-up, according to the guidelines of the national food supplementation programme of Bangladesh. The packets were distributed at discharge from hospital and at each follow-up visit at the CNFU. One packet per day was provided for children below 12 months of age and two packets per day for older children. Food packets were also provided to siblings younger than five years in order to minimise food sharing. Each packet provided about 150 kcal (~ 630 k joules) of energy, with 11% of the energy derived from protein. It contained 20 g of roasted rice powder, 10 g of roasted lentil powder, 5 g of molasses and 3 g of soybean oil [26].

#### Measures

At the end of the third and the sixth month of follow-up children had anthropometric and developmental assessments (BSID-II) at the HNFU [22].

#### Stimulation at home

The quality of stimulation at home was measured by a modified version of the Infant/Toddler HOME inventory [27,28]. The modification was made for use in Bangladesh [29-33]. The phrasing of some of the questions was modified to improve relevance considering differences in living conditions in Bangladesh. The modified version of inventory contained 60 items and six sub-scales: i) Organisation of the physical and temporal environment ii) Stimulation (opportunities for variations in daily stimulation at home) iii) Maternal involvement with the child iv) Play materials v) Avoidance of restriction and punishment and vi) Emotional and verbal responsivity of the mother. The total score was calculated by summing up all positive responses.

A research assistant, unaware of the group assignments, visited the children’s homes and administered the HOME at baseline and after 6 months. Before and during the study, 20 interviews were observed by the trainer and the inter-observer reliability between interviewer and trainer was high (r ranged from 0.91 to 0.98).

##### Child-rearing practices

Research assistants unaware of the group assignment assessed the mother’s child rearing practices at baseline and after 6 months using a modified version of a questionnaire, previously used in Bangladesh [17]. The questions concerned chatting, praising, showing love and affection, how the mothers teached children using different objects or play materials and how they interacted during activities such as feeding, bathing and dressing. We added few items to the parenting questionnaire on setting limits. Total scores were calculated by summing up all positive responses. The test-retest reliability on mothers of 20 children was good (r = 0.92).

##### Maternal depressive symptoms

At baseline and after 6 months the frequency of maternal depressive symptoms was assessed with a questionnaire based on the modified version of the Centre for Epidemiologic Studies-Depression Scale (CES-D) [34]. This instrument was previously used in Bangladesh [35,36] and Jamaica [37,38]. The questionnaire addressed six aspects of depressive symptoms: depressed mood, worthlessness, helplessness/hopelessness, lethargy/fatigue, loss of appetite, and sleep disturbance. The scale was designed to assess the frequency of depressive symptoms expressed in number of days with these symptoms. After piloting, the wording of the questions was adapted to be more culturally appropriate. Mothers were asked to recall how many days in the past week they experienced depressive symptoms and the number of days (0–7) was recorded, and then summed to make a total depression score. Higher scores indicated the presence of more depressive symptoms. Inter-observer reliability between trainer and tester with 20 mothers was high (r = 0.98).

#### Socioeconomic status

Data were collected on the families’ wealth, standard of housing, family structure and parental characteristics.

### Outcomes

The outcomes of this analysis are the quality of home environment (HOME scores) and mothers’ child-rearing practices with the severely malnourished children six months after the start of intervention.

### Sample size

A sample size of 60 in each group was estimated to demonstrate a difference of 5 points in the MDI, based on a previous study in Bangladesh [17] where the MDI had a SD of 12, a significance level of 5% and power of 90% for five treatment groups. There were 59–77 mother-child pairs available for analysis providing 90% power to demonstrate a difference of 0.4 SD in HOME score between any two groups.

### Statistical methods

Data were analysed with SPSS version 18 (SPSS Inc, Chicago). Baseline characteristics were examined of the children and their families among the treatment groups, and of those analysed and lost to follow-up, by analysis of variance (ANOVA) for continuous variables and χ^2^ test for dichotomous variables.

In the analysis of treatment effects across the randomised groups, analysis of covariance (ANCOVA) was used to control for baseline HOME and child rearing practices scores. Adjustment was also made for age at final test and the factors differing among the groups on enrolment and between lost and analysed children, i.e. maternal education and depressive symptoms and father’s occupation.

### Ethics

The proposal was approved by the Institutional Review Board of ICDDR, B. Written informed consent was obtained from mothers on enrolment. Children who did not respond to treatment or had any major illness during the study were treated or referred to health facilities for examination and treatment.

## Results

**Figure 1:**
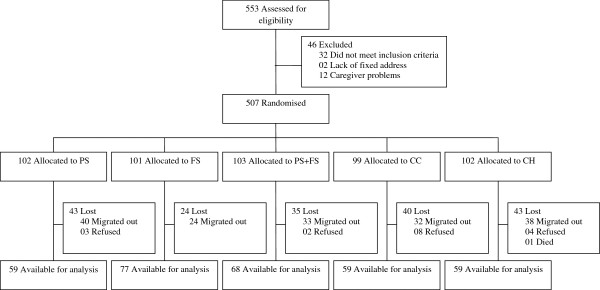
** Study profile. **Flow chart of the sample enrolled and finally analysed. We assessed a total of 553 children for eligibility and enrolled 507 children in the randomised trial. A total of 185 children were lost before applying the final assessment leaving 322 children available for analysis.

Out of 507 children enrolled in the randomised trial, 185 children (36%) were lost before the final assessment leaving a total of 322 children available for analysis (59 children in CH, CC and PS groups, 77 in FS group, and 68 in PS + FS group) (Figure 1). The reasons for loss to follow-up were mainly migration out of the residential area (90% of total loss), refusal to make follow-up visits (9%), and one death. There was significantly less attrition in children receiving food supplementation compared to other groups (*p* = 0.029). Children who were lost differed from those who were analysed in mother’s education, father’s occupation and maternal depressive symptoms. Children of mothers with higher education were more frequently lost in the PS + FS group (*p* = 0.003) and children with lower educated mothers were more frequently lost in the CH group (0.057); fathers having unstable jobs were more frequently lost in PS group (*p* = 0.053). Children of mothers with lower depressive symptoms were more frequently lost in the PS group (*p =* 0.031).

### Baseline characteristics

Baseline household and parental characteristics did not differ among the groups in the analysed children (n = 322), except for maternal depressive symptoms where mothers of children of the CH group had lower scores than mothers in the other groups (*p* = 0.01) (Table 1). There were also no initial differences in the HOME and child rearing practices scores among the randomised groups in the analysed samples (Table 1).

**Table 1:** Baseline characteristics of all children tested at the end of the study (N = 322)

	**PS N = 59**		**FS N = 77**		**PS + FS N = 68**		**CC N = 59**		**CH N = 59**	
	**Mean (SD)**	**95% CI**	**Mean (SD)**	**95% CI**	**Mean (SD)**	**95% CI**	**Mean (SD)**	**95% CI**	**Mean (SD)**	**95% CI**
**Household**										
Asset score^1^	6.1 (2.1)	5.5, 6.6	6.2 (2.3)	5.7, 6.7	6.6 (2.3)	6.1, 7.2	6.3 (2.4)	6.0, 7.0	6.6 (2.5)	6.1, 7.2
Crowding index (person/room)	4.1 (1.3)	3.7, 4.6	4.6 (2)	4.3, 5.0	4.7 (1.6)	4.3, 5.1	4.6 (1.7)	4.2, 5.0	4.4 (1.5)	4.0, 4.8
Housing score^2^	14.0 (2.1)	13.4, 15.0	14.2 (2)	13.7, 14.6	14.2 (2.1)	13.7, 14.7	14.1 (2.2)	13.6, 14.7	14.2 (2.3)	13.6, 14.7
**Parents characteristics**										
Mothers’ age	23.7 (4.5)	22.3, 25.2	23.4 (5.8)	22.2, 24.7	24.6 (6.5)	22.3, 25.2	24.4 (6.1)	23.0, 25.8	23.6 (4.8)	22.1, 25.0
Mothers’ BMI (enrolment)	19.5 (3.1)	18.7, 20.3	19.8 (3.3)	19.1, 20.5	19.2 (2.6)	18.4, 20.0	19.8 (3.2)	19.0, 20.5	19.6 (2.6)	18.8, 20.4
Mothers’ schooling (years)	3.6 (3.6)	2.7, 4.5	3.4 (3.3)	2.6, 4.2	3.1 (3.2)	2.3, 4.0	3.9 (3.8)	3.0, 4.8	4.0 (3.5)	3.1, 4.9
Fathers’ schooling (years)	3.9 (4.0)	2.8, 5.0	4.2 (4.0)	3.2, 5.1	4.7 (3.9)	3.7, 5.7	5.4 (4.8)	4.3, 6.4	5.0 (3.9)	3.9, 6.0
^a^ Mother unemployed (%)	86.4		82.0		82.4		80.0		76.3	
^a^ Father with unstable job (%)	42.4		54.0		55.2		39.0		51.0	
Maternal Depressive symptoms^*3*^ (total days of symptoms)	60.2 (22.4)	54.4, 66.0	58.0 (23.1)	53.0, 63.1	54.9 (20.1)	50.0, 60.4	62.6 (24.6)	57.0, 68.6	49.1 (22.9)	43.3, 55.0
**Total HOME score**^4^	82.5 (6.7)	80.8, 84.1	80.0 (5.7)	78.6, 81.4	81.7 (6.3)	80.2, 83.3	82.7 (6.7)	81.0, 84.3	82.1 (5.7)	80.4, 83.7
**Total child-rearing practices**^5^	25.7 (3.9)	24.8, 26.7	25.1 (3.4)	24.2, 25.9	26.4 (4.0)	25.5, 27.2	26.2 (3.2)	25.2, 27.2	25.7 (3.9)	24.7, 26.6

Analysis of Variance.

^a^ Chi-Square test.

Abbreviations: PS = psychosocial stimulation; FS = food supplementation; PS + FS = both psychosocial stimulation and food supplementation; CC = clinic control and CH = hospital control.

BMI: Body Mass Index (weight in kg/height in meter^2^), HOME: Home Observation for Measurement of the Environment.

^1^ Score was calculated on the presence of 15 common household possessions with a score of ‘1’ for having any of these items and ‘0’ for not having and then summed.

^2^ Condition of roof, floor and wall of the house, type of latrine and availability of water supply was rated and summed.

^3^ In relation to the modified version of Centre for Epidemiologic Studies Depression Scale (CES-D). Mother experiencing depressive symptoms in the past week and the number of days (0–7) was recorded and summed. A higher score indicated having more depressive symptoms.

^4^ Assessed through Betty Caldwell’s Home Observation for Measurement of the Environment (HOME) inventory scale. Total score was obtained by summing all positive responses.

^5^Assessed through a questionnaire, the score was calculated by summing all positive responses.

No significant differences among the groups in the sample tested at the study end for any variables, except for maternal depressive symptoms (*p* = 0.01).

The mean age (SD) of the mothers was 24 (5.6), and 20% were teenage mothers. The mothers had low education level with 37% lacking any schooling [mean years (SD) of schooling: 3.6 (3.5)]. Most mothers were housewives (82%). The overall family income [median (inter-quartile range)] was 4000 (3000–6000) BDT per month (1 US dollar = 68 BDT during the study period).

The HOME at 6 months post intervention was significantly correlated with the children’s scores on the Bayley Scales MDI (r = 0.25, p < 0.01) and PDI (r = 0.23, p < 0.01) whereas child-rearing practices score was significantly correlated with (MDI r = 0.14, p < 0.05) but not PDI.

### Treatment effect on HOME and Child-rearing practices

#### HOME total score

The PS + FS and PS group benefitted in the total HOME, two HOME subscales and child-rearing practices scores after intervention (Table 2) and the overall group difference was significant (p < 0.001).

**Table 2:** HOME and child-rearing practices after six months of intervention (N = 322)

	**PS**		**FS**		**PS + FS**		**CC**		**CH**	
	**Mean (SD)**	**95% CI**	**Mean (SD)**	**95% CI**	**Mean (SD)**	**95% CI**	**Mean (SD)**	**95% CI**	**Mean (SD)**	**95% CI**
**Total HOME**^**1**^	87.9 (6.3)	85.9, 89.1	83.7 (7.3)	83.2, 85.9	89.1 (6.6)	87.6, 90.6	85.2 (8.0)	82.6, 85.9	85.3 (7.0)	83.5, 86.7
**Sub scales of HOME**										
Organisation of environment	18.9 (1.8)	18.5, 19.3	18.5 (1.7)	18.2, 19.0	18.9 (1.7)	18.4, 19.2	18.8 (1.7)	18.4, 19.2	18.5 (1.8)	18.0, 18.9
Stimulation (opportunities at home)	11.7 (1.5)	11.1, 12.0	11.5 (1.8)	11.3, 12.0	11.9 (1.8)	11.5, 12.3	11.6 (1.9)	11.2, 12.0	11.9 (1.8)	11.5, 12.3
Maternal involvement	17.7 (2.1)	17.2, 18.4	16.0 (2.8)	15.6, 16.6	18.4 (1.8)	17.7, 18.9	16.5 (2.9)	15.8, 17.0	16.2 (2.7)	15.5, 16.8
Play materials	16.8 (2.8)	16.0, 17.2	15.3 (2.5)	15.0, 16.1	16.8 (2.8)	16.3, 17.5	15.6 (2.4)	14.9, 16.1	15.9 (2.4)	15.2, 16.5
Avoidance, restriction and punishment	9.5 (0.6)	9.4, 9.7	9.5 (0.7)	9.3, 9.6	9.7 (0.5)	9.5, 9.8	9.5 (0.5)	9.3, 9.6	9.5 (0.7)	9.3, 9.6
Emotion and verbal response	13.3 (1.7)	12.8, 13.8	13.0 (2.1)	12.6, 13.4	13.6 (1.8)	13.1, 14.0	13.0 (1.9)	12.5, 13.4	13.3 (2.3)	12.8, 13.9
**Total child-rearing practices**^**2**^	33.4 (5.2)	32.5, 34.6	25.8 (3.7)	25.1, 27.0	33.1 (4.0)	31.8, 33.8	26.8 (4.4)	25.3, 27.6	24.9 (4.4)	23.9, 26.1

Analysis of covariance controlling for baseline scores, age at final test, maternal education and depression score at baseline and father’s occupation.

Abbreviations: HOME = Home Observation for Measurement of the Environment Inventory; PS = psychosocial stimulation; FS = food supplementation; PS + FS = both psychosocial stimulation and food supplementation; CC = clinic control; CH = hospital control.

^1^ Assessed through Betty Caldwell’s Home Observation for Measurement of the Environment (HOME) inventory scale. Total score was obtained by summing all positive responses.

^2^Assessed through a questionnaire, the score was calculated by summing all positive responses.

*p* < 0.001 for Total HOME and two subscales, maternal involvement and provision of appropriate play materials, and child-rearing practices.

The PS + FS group improved 4.0 points in total HOME score compared with the CH group, 4.8 points compared with the CCgroup and 4.5 points compared with the FS group (p < 0.001 for all). The PS group improved 2.4 points more than the CH group (p = 0.035), 3.3 points more than the CC group (p = 0.004), and 2.9 points more than the FS group (p = 0.006). The above effect sizes varied from 0.66 to 0.33 SD.

#### HOME subscales

The PS + FS and PS groups were significantly different from the other groups (p < 0.001 for all) in two of six HOME sub-scales: maternal involvement and play materials.

##### Maternal involvement

The PS + FS group benefitted 2.2 points in ‘Maternal involvement’ compared with the CH group, 2.1 points compared with the CC group and 2.2 points compared with the FS group (p < 0.001 for all). The PS group benefitted 1.6 points compared with the CH group (p = 0.001), 1.5 points compared with the CC group (p = 0.001), and 1.6 points higher compared with the FS group (p < 0.001). The effect sizes of these differences varied from 0.8 to 0.55 SD.

##### Play materials

The PS + FS group increased 1.2 points in ‘Play materials’ more than the CH group (p = 0.007), 1.6 points more than the CC group (p = 0.001) and 1.5 points more than the FS group (p < 0.001). The PS group increased 1.2 points more than the CC group (p = 0.009) and 1.2 points more than the FS group (p = 0.007). The effect sizes varied from 0.46 to 0.6 SD. There was no significant difference between the PS and CH group.

#### Mothers’ child-rearing practices

Child-rearing practice scores of the PS + FS group improved 7.7, 6.4 and 6.6 points and the PS group improved 8.5, 7.2 and 7.4 points more than the CH, CC and FS groups respectively (p < 0.001 for all). The effect sizes varied from 1.5 to 1.1 SD (Table 3).

**Table 3:** Comparison of child rearing practices score of PS + FS and PS groups with others (N = 322)

	**HOME score**			**Child-rearing practices**		
	**Difference of scores**	**95% CI**	**P**	**Difference of scores**	**95% CI**	**P**
**Combined intervention group (PS + FS)**						
PS + FS vs. CH	4.0	1.8 to 6.1	<0.001	7.7	6.2 to 9.2	<0.001
PS + FS vs. CC	4.8	2.6 to 7.0		6.4	4.8 to 7.9	
PS + FS vs. FS	4.5	2.4 to 6.5		6.6	5.2 to 8.0	
**Stimulation alone group (PS)**						
PS vs. CH	2.4	0.2 to 4.6	0.035	8.5	7.0 to 10.0	<0.001
PS vs. CC	3.3	1.0 to 5.5	0.004	7.2	5.6 to 8.8	
PS vs. FS	2.9	0.8 to 5.0	0.006	7.4	6.0 to 8.9	

Analysis of covariance controlling for baseline scores, age at final test, maternal education and depression score at baseline and father’s occupation.

Abbreviations: PS = psychosocial stimulation; FS = food supplementation; PS + FS = both psychosocial stimulation and food supplementation; CC = clinic control; CH = hospital control.

#### Unfavourable events

The intervention did not include any invasive procedures and no adverse events of the interventions were reported. One child in the CH group died from measles at home.

## Discussion

A short-term community-based intervention with severely malnourished children including parenting education with play activities and the provision of toys and books with or without food supplementation was associated with a marked improvement in quality of home environment and mothers’ parenting practices.

The HOME inventory is a commonly used and validated instrument for the assessment of the quality of home environment and was significantly related to the children’s mental and motor scores on the Bayley Scales. It has been used previously in Bangladesh [29-33]. When implemented in a rural area, HOME scores were related to children’s scores on the Bayley Scales as well as a language test [39]. Another study reported satisfactory internal consistency of the scale (α = 0.72) [31].

In the present study, scores of the total HOME and two of the HOME subscales- ‘Maternal involvement’ and ‘Play materials’ were significantly improved by the PS + FS and PS interventions but not by FS alone. This finding is encouraging, because in the intervention we particularly focused on improving maternal-child interaction.

A substantial number of studies have reported that parenting education has resulted in improved results in subscales of the HOME, especially maternal involvement, play materials, and emotional and verbal responsivity subscales [40]. In Jamaica, mothers with preterm low birth weight infants had higher scores on the total HOME and two specific HOME subscales ‘Avoidance of restriction and punishment’ and ‘Maternal involvement’ [14,41]. Bangladeshi mothers in a rural community-based parenting programme obtained higher scores on the total HOME and on the “Stimulation subscale” (sum of 14 items from the ‘Play materials’ and ‘Maternal involvement’ subscales) than control mothers [16].

Our intervention did not affect the other subscales of the HOME i.e. physical environment, stimulation, and emotional and verbal responsivity. All the families in the present study came from a low socioeconomic background, were dwelling in the slums and had few entertainment facilities at home. However, the intervention was not home-based and did not aim at improving physical conditions of the home beyond efforts to increase safety. Therefore, we did not expect improvements in the ‘Physical environment’ and ‘Stimulation’ but lack of effect on ‘Emotional and verbal responsivity’ was not predicted.

There are relatively few studies in low-income countries to which the results could be compared. The intervention in the present study (sessions every second week through existing primary health care services) had a relatively low intensity in comparison with earlier trials. A meta-analysis of 48 published articles from different socio-economic groups in high-income countries revealed that interventions using 5 to 16 home-based sessions with the mother within a limited period, starting prenatally or 6 months after birth, were the most effective in improving the HOME scores [40].

In this study we reported the parenting practices of mothers who were young, had poor educational levels and were mostly housewives. The opportunities provided for these mothers to practice with their own children probably made the difference. If the mothers’ behaviour is changed, the benefits to their children’s development are more likely to be sustainable [14].

There was considerable attrition from the trial. It is unusual that children with more educated mothers and with less depression were more likely to be lost in the PS + FS and PS groups respectively. It may be that the most disadvantaged families valued the intervention the most. Children with fathers with unstable jobs were also more frequently lost in the PS group. These losses could have potentially modified the effect of the intervention. We therefore employed a conservative approach, controlling for the differences between lost and tested participants as well as any difference among the groups on enrolment in the analysis.

## Conclusions

The results indicate that the home environment and child-rearing practices of mothers of severely malnourished children can be improved through community-based parenting education and play activities with or without food supplementation. For a sustainable programme of psychosocial intervention, mothers/caregivers need to be empowered through training and supervision so they become confident and can carry out the programme activities independently. Further studies in different settings are required to identify the best strategies for supporting parents in caring for their young children in low-income countries.

## Abbreviations

PS: Psychosocial stimulation; FS: Food supplementation; PS + FS: Both psychosocial stimulation and food supplementation; CC: Clinic control; CH: Hospital control; HOME: Home Observation for Measurement of the Environment; SD: Standard deviation; ICDDR,B: International Centre for Diarrhoeal Disease Research, Bangladesh; MDI: Mental development index; BSID: Bayley Scales of Infant Development; WAZ: Weight-for-age Z-score; WLZ: Weight-for-length Z-score; CNFU: Community nutrition follow-up unit; HNFU: Hospital nutrition follow-up unit; PL: Play leader; UNICEF: United Nation Children’s Education Fund; CESD: Centre for Epidemiologic Studies-Depression Scale; ANOVA: Analysis of variance; ANCOVA: Analysis of co-variance; BDT: Bangladeshi taka.

## Competing interests

The authors declare that they have no competing interests.

## Author’s contributions

BN was responsible for proposal writing, field supervision, and data analysis and reporting of the study results. IH designed the community-based management with food supplementation and supervised field visits. JH supervised field visits, read the first draft and made suggestions. TA was one of the co-supervisors, assisted with the study design and edited the first draft. SM was another co-supervisor, designed the stimulation intervention, read the first draft and edited. LA was the supervisor and assisted with data analysis, manuscript writing and editing of the drafts. All authors read and approved the final manuscript.

## Pre-publication history

The pre-publication history for this paper can be accessed here:

http://www.biomedcentral.com/1471-2458/12/622/prepub
